# Etiological and clinical characteristics of severe pneumonia in pediatric intensive care unit (PICU)

**DOI:** 10.1186/s12887-023-04175-y

**Published:** 2023-07-15

**Authors:** Dongmei Chen, Lu Cao, Wenjing Li

**Affiliations:** 1 Department of Emergency, Children’s Hospital of Nanjing Medical University, Nanjing, China; 2 Department of Pharmacy, Children’s Hospital of Nanjing Medical University, Nanjing, China

**Keywords:** Severe pneumonia, Pediatric intensive care unit, Pathogenic characteristics

## Abstract

**Objective:**

To analyze the etiological distribution characteristics of pediatric patients with severe pneumonia admitted to the Pediatric Intensive Care Unit (PICU), in order to provide a reference for the rational use of clinical antimicrobial drugs.

**Methods:**

A retrospective analysis of pediatric patients admitted to PICU with a diagnosis of severe pneumonia from January 2018 to December 2021 was performed and statistical analysis of pathogenic characteristics was performed.

**Results:**

A total of 649 pathogens were detected in 515 children, with a positive detection rate of 77.48%. Bacteria were detected at the highest rate (40.52%), followed by viruses (34.35%), atypical pathogens (19.72%) and fungal (4.31%). Gram-positive infections were dominated by Staphylococcus aureus (39.56%) and Streptococcus pneumoniae (32.97%), and Gram-negative infections were dominated by Acinetobacter Bahmani (16.28%) and Haemophilus influenzae (15.12%), followed by Klebsiella pneumoniae (13.95%) and Pseudomonas aeruginosa (12.21%). Viral infections were dominated by respiratory syncytial virus (25.65%) and EB virus (20.43%), fungal infections were dominated by Candida albicans (50.0%). The proportion of children infected with single pathogen (49.62%) was comparable to that of those with mixed infections (50.38%). There were statistically significant differences in the distribution of children with single pathogen infection by gender (P < 0.05). The age distribution of children with single bacterial, single viral and single fungal infections was statistically different (P < 0.05). There was no significant difference in the distribution of onset season in children with single pathogen infections (P > 0.05), but the number of children with single viral infections was significantly higher in winter and spring than that in summer and autumn, and the difference was statistically significant (P < 0.05). A mixture of 2 pathogens (77.61%) accounted for the majority of mixed infections, there were statistical differences in the distribution of bacterial + viral infection in terms of gender, age, and onset season (P < 0.05), children with viral + mycoplasma infection in terms of gender and age (P < 0.05), and children with viral + fungal infection in terms of gender (P < 0.05), and children with bacterial + mycoplasma infection in terms of age and onset season (P < 0.05). Among the children infected with 3 pathogens, there were statistically significant differences in the distribution of bacterial + viral + fungal and viral + mycoplasma + fungal infections in terms of gender (P < 0.05), and children with bacterial + viral + mycoplasma infection in terms of age (P < 0.05), while there was no significant difference in the distribution of onset season (P > 0.05). There were no significant differences in the distribution of children infected with 4 pathogens in terms of gender, age and onset season (P > 0.05).

**Conclusion:**

The pathogens of pediatric patients with severe pneumonia in PICU commonly involves bacteria and viruses. As the age of children grows, the detection rate of bacteria shows a decreasing trend, and the pathogenic spectrum gradually changes from bacteria to mycoplasma and viruses, and the number of mixed infections gradually increase. Rational selection of antimicrobial drugs needs to consider pathogenic characteristics of different age, gender, and onset season in clinical practice.

## Introduction

Severe pneumonia (SP) is a complex syndrome of lung infections [1] which may be defined by the clinical manifestations, extent of pulmonary lesions, presence of hypoxemia, and manifestations of pulmonary and extrapulmonary complications. If there is general deterioration, refusal to eat or signs of dehydration, altered consciousness, significantly increased respiratory rate (infants: >70 breaths per minute or older children: >50 breaths per minute), central cyanosis, respiratory distress (grunting, nasal flaring, chest indrawing), involvement of multiple lung lobes or more than two-thirds of one lung, pleural effusion, pulse oxygen saturation ≤ 0.92 (at sea level), or any other extrapulmonary complications, the presence of any one of these criteria is considered as SP [2, 3].

SP remains the leading cause of death in children [4]. Each year, an estimated 12 to 15.6 million children under 5 years old are affected by pneumonia, leading to approximately 1 million children fatalities [5]. Additionally, it is observed that around 1.4 million cases of pneumonia progress to SP [6]. Children at different age groups exhibit varying susceptibilities and outcomes to SP. Infants and very young children, with their developing immune systems and smaller airways, may be more susceptible to SP [7]. Older children may also experience differences in susceptibility based on their immune maturity and exposure to environmental risk factors [8]. Additionally, certain age-specific risk factors, such as attending daycare or school, may contribute to the transmission and severity of pneumonia in children [9]. Furthermore, gender and season may play a role in the etiology of SP in children. Certain seasons, such as winter, are commonly associated with increased respiratory infections, including SP [10]. Factors such as changes in temperature, humidity, viral circulation patterns, and indoor crowding during different seasons can contribute to the incidence of SP in children [6, 11]. There may be differences in the susceptibility of SP between male and female children. These differences could be attributed to various factors, including hormonal variations, genetic predispositions, anatomical differences, immune response variations, or variances in exposure to risk factors [12]. For example, previous studies have indicated that certain respiratory infections may exhibit a higher prevalence or severity in male children due to differences in immune response or behavioral factors [13]. In summary, gender, age and seasons collectively impact the etiology of SP in children, and we acknowledge that further research and analysis are necessary to thoroughly investigate and validate the associations in the pediatric population.

The pediatric intensive care unit (PICU) serves as a crucial facility for rescuing, treating, and caring for critically ill children, including those with SP. Due to the severity of their illness and potential immune deficiency, children admitted to the PICU are at a higher risk of cross-infection. Prolonged hospitalization and invasive procedures further contribute to the increased likehood of cross-infection. The widespread use of antibiotics, especially broad-spectrum antibiotics, has also led to the emergence of drug resistant pathogens over time. Consequently, early, timely and accurate identification of the causative pathogens, along with the appropriate selection of antimicrobial drugs, is vital for improving outcomes, reducing drug resistance, and lowering mortality rates in children. In our study, we conducted a retrospective analysis of pathogenic datafrom515 children hospitalized in the PICU with SP between January 2018 and December 2021. By investigating the changing pathogenic spectrum and identifying factors such as gender, age and seasons, we aimed to analyze the etiological characteristics and clinical features of SP in children, highlighting the need for appropriate diagnostic and therapeutic strategies in managing SP cases in PICU and providing valuable insights for the rational use of antimicrobial drugs in the PICU.

## Materials and methods

### Subjects

A retrospective study was conducted to collect data on the treatment of pediatric patients hospitalized in PICU with a diagnosis of SP from January 2018 to December 2021. The etiological results of the first culture were collected for analysis.

Inclusion Criteria: (1) Children who met the diagnostic criteria for SP, including community acquired pneumonia; (2) Pediatric patients aged > 29 days admitted to the PICU;

Exclusion Criteria:(1) Patients with incomplete medical records; (2) Patients whose parents refused to cooperate in completing relevant pathogenic examinations were excluded; (3) Patients with immunocompromised were excluded; (4) Patients diagnosed with SP due to nosocomial infection or ventilator-associated pneumonia were excluded; (5) Patients with culture results determined to be contaminating or colonizing bacteria were excluded.

Patients with immunocompromised refer to children whose immune system is impaired, weakened, or compromised, as evidenced by abnormal results in immunological function tests in our study. This impairment leads to an increased susceptibility to infections and a reduced ability to mount an effective immune response.

### Clinical information

Clinical data including basic information about the child: age, gender, weight, diagnosis, duration of hospitalization;

The relevant pathogenic examinations include bacterial culture, respiratory viral detection, mycoplasma pneumoniae detection, and fungal tests. Specimens were collected from patients within the first 24 h of admission, and all specimens collected were processed using specific reagent kits following the manufacturer’s instructions. Sputum and blood cultures were obtained as the primary sources for bacterial culture, with deep sputum samples obtained through airway suction accounting for the majority (76.35%) of collected specimens. The direct immunofluorescence assay is used to detect common respiratory viruses in sputum samples, such as respiratory syncytial virus, human metapneumovirus, influenza A virus, influenza B virus, and adenovirus. The proportion of virus detection in blood samples using respiratory virus IgM antibody testing is similar to that in sputum samples. The detection of mycoplasma pneumoniae infection involves measuring IgM antibodies using an indirect immunofluorescence assay. Fungal detection is primarily determined through sputum culture. For patients with high-risk factors for deep fungal infections, the(1,3) - β-D-glucan assay and semiquantitative mannan tests were employed for detection, which account for only 7.21% of cases.

### Statistical analysis

SPSS 26.0 software was used for data analysis, and the count data were analyzed by descriptive statistics and expressed as percentages (%) or rates, and χ^2^ test was used for comparison of etiological distribution characteristics of children with SP between groups such as different ages, genders and seasons, and the difference was considered statistically significant at P < 0.05.

We classified the patients into five age groups according to the text Pediatrics: < 1 year, 1–3 years, 4–7 years, 8–14 years, and 15–18 years [14]. We stratified each year into four seasons, such as spring (from March to May), summer (from June to August), autumn (from September to November) and winter (from December to February).

## Results

### General information

From January 2018 to December 2021, a total of 515 pediatric patients diagnosed with severe pneumonia in PICU, including 294 males (57.09%) and 221 females (42.91%), with a male to female ratio of 1.33:1. Among which 279 cases (54.18%) were < 1 years old, which accounted for the largest proportion, followed by 137 cases (26.60%) of 1-3 years old, 55 cases (10.68%) of 4-7 years old, 43 cases (8.35%) of 8-14 years old, and only 1 case (0.19%) of > 14 years old, with significant differences in the age distribution of children (*P* < 0.05). The number of the children affected was 140 (27.19%), 112 (21.75%), 116 (22.52%) and 147 (28.54%) in spring, summer, autumn and winter, respectively, with a high incidence in winter and spring. However, there was no significant difference in onset season (P > 0.05).

### Analysis of etiological results

#### Overall detection analysis

A total of 649 pathogens were detected in 515 children, of which 263 (40.52%) were bacteria, including 172 (65.40%) of gram-negative bacteria (G-) and 91 (34.60%) of gram-positive bacteria (G+);230 (35.44%) of viruses, with 79 (34.35%) of respiratory syncytial virus predominating; 128 (19.72%) of atypical pathogens, dominated by 102 (79.69%) of mycoplasma, 20 (15.62%) and 6 (4.69%)of UU and CT, respectively; 28 (4.31%) of fungus, dominated by 14 (50.0%) of candida albicans, followed by 5 (17.86%) of other candida and 3 (10.71%) of candida tropicalis.

According to the statistics of detection year, 228, 287, 73, and 61 pathogens were detected from 2018 to 2021, respectively, and the number of detection showed a decreasing trend year by year, but the percentage of bacteria, viruses, mycoplasma and fungus detected was not significantly different in each year (P > 0.05); There were no significant differences in the gender distribution of the positive results for the detected pathogens (P > 0.05). There was no significant difference in the distribution of positive pathogens between years for children < 1 year old and 1-3 years old (P > 0.05), but there was a significant difference in the distribution of positive pathogens between years for children 4-7 years old and 8-14 years old (P < 0.05), the proportion of positive pathogens detected in 2020 and 2021 was significantly higher than that in 2018 and 2019. The proportion of positive pathogens detected in spring was not significantly different between years (P > 0.05), but the proportion of positive pathogens detected in summer, autumn and winter was significantly different between years (P < 0.05), with the proportion of positive pathogens detected in summer and autumn decreasing significantly in 2020, while the proportion of positive pathogens detected in winter increased significantly in 2020. The results are shown in Table 1.

Of the 515 children, 399 were able to be identified infected, with a positive pathogen detection rate of 77.48%. There were 198 cases (49.62%) of single pathogen infection and 201 cases (50.38%) of mixed infection. Among the 201 children with mixed infection, mixed infection with 2 pathogens predominated with 156 cases (77.61%), followed by 41 cases (20.40%) of 3 pathogens and 4 cases (0.78%) of 4 pathogens, respectively. There were 6 types of mixed infection of 2 pathogens, among which, mixed infection of bacterial + virus and virus + mycoplasma accounted for a relatively high proportion, 80 cases (51.28%) and 40 cases (25.64%), respectively, followed by mixed infections of bacterial + mycoplasma in 27 cases (17.31%), and mixed infections of bacterial + fungus, virus + fungus and mycoplasma + fungus accounted for relatively lower proportion, 6 cases (3.85%), 2 cases (1.28%) and 1 cases (0.64%), respectively; There were 4 types of mixed infection of 3 pathogens, of which 28 cases (68.29%) were mixed infections of bacterial + virus + mycoplasma, accounting for a relatively higher proportion, followed by 10 cases (24.39%) mixed infections of bacterial + virus + fungus, 1 case (2.44%) mixed infections of bacteria + mycoplasma + fungus and 2 cases (4.88%) mixed infections of virus + mycoplasma + fungus, respectively; There were only 4 cases of children with mixed infection of 4 pathogens, and all of whom were hospitalized for more than 2 weeks.

**Table 1:** Overall detection of positive pathogenic results [number of cases (%)]

Characteristics	2018	2019	2020	2021	*P*-value*
	228(100.00)	287(100.00)	73(100.00)	61(100.00)	< 0.001
Pathogenic species, n (%)					
Bacteria	85(37.28)	112(39.02)	30(41.10)	36(59.02)	0.285
Virus	84(36.84)	105(36.59)	26(35.61)	15(24.59)	0.616
Mycoplasma	53(23.25)	55(19.16)	14(19.18)	6(9.83)	0.265
Fungus	6(2.63)	15(5.23)	3(4.11)	4(6.56)	0.447
Gender, n (%)					
Male	132(57.89)	182(63.41)	36(49.32)	28(45.90)	0.462
Female	96(42.11)	105(36.59)	37(50.68)	33(54.10)	0.293
Age (years), n (%)					
< 1 year	130(57.02)	141(49.13)	33(45.21)	27(44.26)	0.600
1–3 years	67(29.39)	98(34.15)	18(24.66)	15(24.59)	0.528
4–7 years	16(7.02)	39(13.59)	15(20.55)	11(18.03)	0.021
8–14 years	15(6.58)	9(3.14)	7(9.59)	8(13.11)	0.020
15–18 years	0(0)	0(0)	0(0)	0(0)	-
Seasons, n (%)					
Spring	59(25.88)	74(25.78)	9(12.33)	21(34.43)	0.118
Summer	41(17.98)	64(22.30)	4(5.48)	11(18.03)	0.042
Autumn	78(34.21)	50(17.42)	6(8.22)	20(32.79)	< 0.001
Winter	50(21.93)	99(34.49)	54(73.97)	9(14.75)	< 0.001

Data are presented as number (%)

#### Analysis of the distribution of children with positive pathogenic results by gender

The gender distribution of 399 children with identified infections was analyzed and the results are shown in Table 2. There were more boys than girls with single pathogen infection, with a statistically differences in gender distribution (P < 0.05), and children with single bacterial and single viral infections had a statistically significant difference in gender distribution (P < 0.05). Among children with the 2 pathogenic infections, there were statistically significant differences in the gender distribution of children with bacteria + virus, virus + mycoplasma and virus + fungal infection (P < 0.05), while there were no significant gender differences in the other three modes of infection (P > 0.05); Among the children with the 3 pathogenic infections, there were significantly more boys than girls with bacterial + virus + fungal infections, and there were statistically significant differences in the gender distribution (P < 0.05), and there were statistically significant differences in the gender distribution for children with virus + mycoplasma + fungal infection (P < 0.05), while there was no significant gender differences for the other 3 modes of infection (P > 0.05); There was no significant difference in the gender distribution of children infected with the 4 pathogens (P > 0.05).

**Table 2:** Gender distribution of 399 children with positive pathogenic results [number of cases (%)]

Gender	Single pathogen infection					2 pathogenic infections							3 pathogenic infections					4 pathogenic infections
	BAT	VIR	MP	F	Sum	BAT + VIR	VIR + MP	BAT + MP	BAT + F	VIR + F	F + MP	Sum	BAT + VIR + MP	BAT + VIR + F	BAT + MP + F	VIR + NP + F	sum	BAT + VIR + MP + F
Male	60(56.07)	39(61.90)	14(53.85)	1(50.0)	114	47(58.75)	26(65.0)	13(48.15)	3(50.0)	2(100.0)	0(0)	91	13(46.43)	10(100.0)	1(100.0)	2(100.0)	26	1(25.0)
Female	47(43.93)	24(38.10)	12(46.15)	1(50.0)	84	33(41.25)	14(35.0)	14(51.85)	3(50.0)	0(0)	1(100.0)	65	15(53.57)	0(0)	0(0)	0(0)	15	3(75.0)
Sum	107	63	26	2	198	80	40	27	6	2	1	156	28	10	1	2	41	4
P	0.076	0.008	0.579	1.000	0.003	0.027	0.007	0.785	1.000	0.046	0.157	0.003	0.593	< 0.001	0.157	0.046	0.01	0.157

Data are presented as number (%)

BAT, Bacteria; VIR, Virus; MP, Mycoplasma; F, Fungus;

#### Analysis of the distribution of children with positive pathogenic results by age

Among the 399 children with identified infected pathogens, there were no children > 14 years old, and the age distribution of children with detected pathogens was analyzed, and the results are shown in Table 3. Among children with single pathogen and mixed pathogen infections, the highest proportion of children < 1 year old; Among the children with single pathogen infections, there were only 2 children < 1 year old with single fungal infections, and there were statistically significant differences in the age distribution of children with single bacteria, single viral and single fungal infection (P < 0.05). Among children with 2 pathogens, there were statistically significant differences in the age distribution of children with bacterial + viral, viral + mycoplasma and bacterial + mycoplasma infection (P < 0.05), while there were no significant differences in the age distribution of children with bacterial + fungal, viral + fungal and mycoplasma + fungal infection (P > 0.05); Among the children with 3 pathogens, there was a statistically significant difference in the age distribution of children with bacterial + viral + mycoplasma infections (P < 0.05), and there was no significant difference in the age distribution of the other 3 modes of infection(P > 0.05). There was no significant difference in the age distribution of children with 4 pathogens (P > 0.05).

**Table 3:** Age distribution of 399 children with positive pathogenic results [number of cases (%)]

Age (year)	Single pathogen infection						2 pathogenic infections						3 pathogenic infections					4 pathogenic infections
	BAT	VIR	MP	F	Sum	BAT + VIR	VIR + MP	BAT + MP	BAT + F	VIR + F	MP + F	Sum	BAT + VIR + MP	BAT + VIR + F	BAT + MP + F	VIR + MP + F	Sum	BAT + VIR + MP + F
< 1	72(67.29)	35(55.56)	8(30.77)	2(100.0)	117	52(65.0)	12(30.0)	14(51.85)	3(50.0)	1(50.0)	0(0)	82	9(32.14)	5(50.0)	0(0)	0(0)	14	2(50.0)
1-3	22(20.56)	21(33.33)	7(26.92)	0(0)	50	21(26.25)	13(32.5)	9(33.33)	2(33.33)	0(0)	0(0)	45	14(50.0)	3(30.0)	0(0)	1(50.0)	18	1(25.0)
4-7	6(5.61)	5(7.94)	7(26.92)	0(0)	18	6(7.50)	15(37.5)	1(3.70)	0(0)	1(50.0)	1(100.0)	24	4(14.29)	1(10.0)	0(0)	0(0)	5	0(0)
8-14	7(6.54)	2(3.17)	4(15.38)	0(0)	13	1(1.25)	0(0)	3(11.11)	1(16.67)	0(0)	0(0)	5	1(3.57)	1(10.0)	1(100.0)	1(50.0)	4	1(25.0)
Sum	107	63	26	2	198	80	40	27	6	2	1	156	28	10	1	2	41	4
P	< 0.001	< 0.001	0.605	0.046	< 0.001	< 0.001	< 0.001	< 0.001	0.217	0.446	0.261	< 0.001	< 0.001	0.118	0.261	0.446	< 0.001	0.446

Data are presented as number (%)

BAT, Bacteria; VIR, Virus; MP, Mycoplasma; F, Fungus;

#### Analysis of the distribution of children with positive pathogenic results by season

The distribution of 399 children with identified pathogens was analyzed according to the season of onset, and the results are shown in Table 4. Among children with single pathogen infections, the number of children with single viral infection was significantly higher in winter and spring than in summer and autumn, and the difference was statistically significant (P < 0.05), and there was no significant difference in the distribution of the onset season of children with single pathogen infections (P > 0.05); Among children with 2 pathogens, children with mixed bacterial + viral infections had a higher incidence in winter, followed by spring, summer and autumn, with statistically significant differences in the distribution of the onset season (P < 0.05), and children with mixed bacterial + mycoplasma infections were higher in autumn, with statistically significant differences in the distribution of the onset season (P < 0.05), while there was no significant difference in the other 4 nodes of infection by season(P > 0.05). There were no significant differences in the distribution of the onset season of children with 3 and 4 pathogenic infections (P > 0.05).

**Table 4:** Seasonal distribution of 399 children with positive pathogenic results [number of cases (%)]

Season	Single pathogen infection						2 pathogenic infections						3 pathogenic infections					4 pathogenic infections
	BAT	VIR	MP	F	Sum	BAT + VIR	VIR + MP	BAT + MP	BAT + F	VIR + F	MP + F	Sum	BAT + VIR + MP	BAT + VIR + F	BAT + MP + F	VIR + MP + F	Sum	BAT + VIR + MP + F
Spring	27(25.23)	17(26.98)	7(26.92)	0(0)	51	20(25.0)	9(22.5)	7(25.93)	0(0)	1(50.0)	0(0)	37	6(21.43)	3(30.0)	0(0)	1(50.0)	10	2(50.0)
Summer	28(26.17)	10(15.87)	3(11.54)	1(50.0)	42	12(15.0)	10(25.0)	5(18.52)	2(33.33)	0(0)	1(100.0)	30	4(14.29)	2(20.0)	0(0)	0(0)	6	0(0)
Autumn	25(23.36)	11(17.46)	8(30.77)	1(50.0)	45	12(15.0)	12(30.0)	12(44.44)	2(33.33)	1(50.0)	0(0)	39	8(28.57)	1(10.0)	0(0)	0(0)	9	1(25.0)
Winter	27(25.23)	25(39.68)	8(30.77)	0(0)	60	36(45.0)	9(22.5)	3(11.11)	2(33.33)	0(0)	0(0)	50	10(35.71)	4(40.0)	1(100.0)	1(50.0)	16	1(25.0)
Sum	107	63	26	2	198	80	40	27	6	2	1	156	28	10	1	2	41	4
P	0.971	0.007	0.322	0.446	0.165	< 0.001	0.849	0.032	0.446	0.446	0.261	0.071	0.283	0.446	0.261	0.446	0.076	0.446

Data are presented as number (%)

BAT, Bacteria; VIR, Virus; MP, Mycoplasma; F, Fungus;

#### Distribution of pathogenic infections in children

A total of 39 bacteria were detected in 399 children with identified pathogens, including 12 gram-positive bacteria (G+) and 27 gram-negative bacteria (G-), with 91 and 172 strains, respectively; The number of virus species detected was 10, with 230 positive results; A total of 128 positive results for atypical pathogens were detected, including 102 positive results for mycoplasma; The number of fungal species detected was 8, with 28 positive results. The types and proportion of pathogens detected are shown in Table 5. The top three pathogens detected in children with bacterial infection were G + Staphylococcus aureus, Streptococcus pneumoniae and Staphylococcus epidermididis, G-Acinetobacter baumannii, Haemophilus influenzae and Klebsiella pneumoniae, and the top three viruses detected were respiratory syncytial virus, EB Viruses and adenoviruses, and the top three fungus detected were Candida albicans, other Candida albicans, and Candida tropicalis.

**Table 5:** Composition distribution of pathogen detection results [Number of detection (%)]

Sorts	Bacteria				Virus		Fungus	
	G+	n(%)	G-	n(%)	Virus	n(%)	Fungus	n(%)
1	Staphylococcus aureus	36(39.56)	Acinetobacter baumannii	28(16.28)	Respiratory syncytial virus	79(34.35)	Candida albicans	14(50.0)
2	Staphylococcus pneumoniae	30(32.97)	Haemophilus influenzae	26(15.12)	Epstein-Barr virus	47(20.43)	Other Candida	5(17.86)
3	Staphylococcus epidermidis	9(9.89)	Klebsiella pneumoniae	24(13.95)	Adenovirus	30(13.04)	Candida tropicalis	3(10.71)
4	Staphylococcus hominis	7(7.69)	Pseudomonas aeruginosa	21(12.21)	Cytomegalovirus	26(11.30)	Filamentous fungi	2(7.14)
5	Staphylococcus capitis	2(2.20)	Escherichia coil	14(8.14)	Human parainfluenza virus	24(10.43)	Candida parapsilosis	1(3.57)
6	Staphylococcus haemolyticus	2(2.20)	Enterobacter cloacae	10(5.81)	Influenza B virus	15(6.52)	Candida famata	1(3.57)
7	Staphylococcus warneri	2(2.20)	Burkholderia cepacia	7(4.07)	Influenza A virus	10(4.35)	Candida guilliermondii	1(3.57)
8	Enterococcus faecium	1(1.10)	Stenotrophomonas maltophilia	6(3.49)	EV71	7(3.04)	Aspetgillus	1(3.57)
Total	-	91 (100)	-	172 (100)	-	230 (100)	-	28 (100)

Data are presented as number (%)

## Discussion

Children are susceptible to pneumonia and prone to severe pneumonia due to differences in their respiratory anatomy, physiological characteristics and immune function compared to adults [15]. Worldwide, about 7%~13% of pediatric patients diagnosed with pneumonia each year belong to severe pneumonia, and about 2 million children die as a result, making severe pneumonia a serious threat to children’s health. Severe pneumonia is one of the most common critical diseases in PICU, and the current treatment of severe pneumonia is mainly anti-infective treatment for pathogenesis and symptomatic treatment for complications, therefore, early and correct empirical treatment can improve the efficacy of diagnosis and treatment of severe pneumonia, which is the key to influence the prognosis of the disease and reduce the mortality rate. In this article, we retrospectively analyzed the pathogenic results of 515 pediatric patients diagnosed with severe pneumonia who were hospitalized in PICU from January 2018 to December 2021, and conducted subgroup analysis according to gender, different age group and different seasons of onset to explore the common pathogens of severe pneumonia in PICU and the characteristics of pathogenic distribution, aiming to provide a basis for early pathogenic determination and rational empirical anti-infective treatment.

Among the 515 children with severe pneumonia, 399 cases were able to identify the infection, and the positive rate of pathogen detection was 77.48%. Bacteria were the most detected among all the pathogens, and the pathogenic composition ratio according to gender, age and onset season revealed the highest rate of bacterial detection, indicating that severe pneumonia was mainly caused by bacteria, with G- predominating, accounting for 65.40%, which was lower than the statistical results of CHINET from 2005 to 2021, ranged from 65.7 to 73.0% [16]. The possible reason is that most of the children in PICU were referred from other departments or other hospitals, and had received antibiotics treatment before admission to the PICU, which affects the positive rate of specimens to a certain extent and resulting in an increased proportion of false negatives. Acinetobacter baumannii was the highest detected in G-infections, followed by Haemophilus influenzae and Klebsiella pneumoniae, which was different from the results of many PICU [17, 18] in China or the results of our hospital in which the highest G- detection rate was Escherichia coli. Acinetobacter baumannii is widely distributed in the hospital environment and is one of the more common pathogenic bacteria of nosocomial infections [19], which easily causing ventilator-associated pneumonia, bacteremia, meningitis and respiratory infections [20]. Acinetobacter Baumann infections account for 20% of ICU infections worldwide, and the frequency of community-acquired infections has been gradually increased in recent years [21]. Klebsiella pneumoniae is a conditionally pathogenic bacterium, which is highly pathogenic to the human. when human immunity is reduced, and invasive operations such as endotracheal intubation or mechanical ventilation, deep vein catheterization, urinary tract intubation are performed, Klebsiella pneumoniae colonized in the human intestines and respiratory tracts will take the opportunity to invade wounds and cause infections, which will lead to sepsis in severe cases [22, 23]. Most of the children in PICU are critically ill, often accompanied by severe pathophysiological disorders and immune deficiency, and some children are admitted with tracheal intubation or mechanical ventilation, which damage the respiratory barrier and provide a portal for pathogens to enter the respiratory tract directly, increasing the chance of infection. At the same time, the massive use of a variety of broad-spectrum antibiotics alters the normal parasitic flora and causes infection by conditionally pathogenic bacteria. The difference in G-bacteria detection results suggests that a more targeted anti-infective regimen for initial treatment according to the pathogenic composition. The G + detected was dominated by Staphylococcus aureus, followed by Streptococcus pneumoniae and Staphylococcus epidermidis, which was consistent with the most reports [17, 24]. Staphylococcus aureus is highly resistant to the outside world and is one of the main pathogenic bacteria of nosocomial infections and community-acquired infections, among which MRSA is multi-drug resistant and has a high mortality rate once infected, which is called a “superbug” [25, 26], suggesting that children with severe pneumonia in PICU should be alert for mixed G + infections when treating G-infection. Staphylococcus epidermidis colonizes the surface of human skin and mucous membranes, is weakly virulent and was previously considered a non-pathogenic or weakly pathogenic bacteria, but when infecting hosts with specific risk factors, such as implantation of medical devices, which can repurpose factors involved in colonization, resulting in up to 30% of nosocomial bloodstream infections [27]. Therefore, the main high-risk factor for PICU is mechanical assisted ventilation, suggesting that we should pay attention to the control of Staphylococcus epidermidis infection.

Viruses are important pathogen causing severe pneumonia in children, and respiratory syncytial virus has the highest detection rate [28]. The detection rate of viruses in PICU with severe pneumonia in Chongqing, China, was reported as high as 72.3%, of which the highest rate of respiratory syncytial virus detection was 41.2% [29], and in Suzhou, the detection rate of respiratory syncytial virus was reported to be 21.83%, which ranked the first respiratory virus detected [15, 30]. The detection rate of the virus in this study was 35.44%,with the highest rate of 34.35% for respiratory syncytial virus, which is consistent with the literature reports, further confirming that respiratory syncytial virus is an important viral pathogen in children with severe pneumonia.

Mycoplasma is one of the important pathogenic bacteria causing respiratory infections in children, which not only causes inflammatory responses in the respiratory system, but also participate in the immune response at the same time of infection, forming immune complexes in target organs outside the lungs and thus showing the corresponding symptoms, and mycoplasma infection can range from mild pharyngitis, bronchitis to severe pneumonia, even severe infections can lead to death [31]. In recent years, the number of cases of severe pneumonia caused by mycoplasma pneumoniae has increased significantly, and the immune function of the organism, mixed infections, and resistance to macrolide antibiotics may be involved in the development of severe mycoplasma pneumonia. The detection rate of atypical pathogens in this study was 19.72%, of which mycoplasma accounted for 15.72%, which is higher than that of other regions [15, 17].

Fungal infections accounted for 4.35% in this study, mostly in patients with underlying diseases who were admitted to hospital with SP but had normal immunological tests results. These children may be also be susceptible to fungal infections, with Candida albicans being the most common.In addition to single infection, mixed infections accounted for a large proportion (50.38%) of pathogens in children with severe pneumonia in the PICU, and mixed infection with 2 pathogens accounted for the majority (77.61%), with bacterial combined with viral infections being the most common (51.28%). In terms of age distribution, children < 1 year old had the highest proportion of single pathogen infection and 2 pathogen infections, while the number of children with 3 pathogen infections was higher in the age group of 1-3 years than < 1 years old, and the proportion of children with mycoplasma infections and combined mycoplasma infections was significantly higher in the age groups of 1-3 years and 4-7 years, and the proportion of children aged 1-3 years was slightly higher than that of children aged 4-7 years. Children with severe pneumonia in PICU account for 53.88% within 1 year old, probably due to the gradual depletion of IgG brought by the mother after birth and insufficient self-synthesis by themselves; In addition, for children < 1 year old, especially small infants, the normal flora is not well established, the barrier function of the skin and mucosa is low, the ability to produce specific IgM is poor, and the immune function is low and defense function IgA is reduced, the destruction of the adherent protein layer leads to bacterial adhesion on the trachea, the inhalation of colonized bacteria in the oropharynx, gastric reflux is easy to accidentally aspirate into the airway, other invasive operations such as mechanical ventilation, and the use of broad-spectrum antibacterial drugs, hormones and other immunosuppressive drugs are more frequent, viruses and bacteria are easy to invade, and the types of bacteria are diversified are prone to cause multiple infections. However, for children aged 1-7 years, especially those aged 1-3 years, the antibody IgG from the mother is gradually depleted, and the antibody production system is not yet mature, especially IgG2 which is a subclasses of IgG grow very slowly, making young children more susceptible to respiratory infectious diseases as they live more in groups. It can be seen that the bacterial detection rate showed a decreasing trend as the children grew older, and the pathogenic spectrum gradually changed from bacteria to mycoplasma and viruses, with a gradual increase in mixed infections, suggesting that the change in the pathogenic spectrum may be closely related to the developmental process of immune function in children, and the specific mechanism needs to be further explored. In terms of seasonal distribution, both bacteria and viruses were detected most frequently in winter and spring, with the detection rate being higher in winter than in spring, and reaching a peak around January, which is consistent with the seasonal prevalence of severe pneumonia. The wet and cold climate is the most important factor leading to the epidemic of severe pneumonia, especially viral infections, and the study is in an area with a cold and wet winter climate, reaching the coldest temperatures around January each year, so the highest detection of virus. Mycoplasma was detected highest in autumn, followed by winter and spring, which differs from the literature reported a predominant of winter and spring incidence [32]. Among the mixed infections, bacterial + viral infections had the highest detection rate in winter, followed by spring, while mixed fungal infections did not show seasonality due to low detection rates.

In addition, analyzed by statistical year, the overall detection of pathogens in 2020 and 2021 was significantly lower than in 2018 and 2019, probably due to a significant reduction in respiratory morbidity and fewer children with severe pneumonia admitted to the PICU and a consequent decrease in the detection rate of pathogens since the COVID-19 epidemic, due to the implementation of universal masking precautions in China [33]. Notable, for children < 1 and 1-3 years old, pathogen detection rate were lower in 2020 and 2021 than in 2018 and 2019, but for children aged 4-7 and 8-14 years old, pathogen detection rates were higher in 2020 and 2021 than that in 2018 and 2019, and the differences were statistically significant (P < 0.05). The seasonal distribution showed that the pathogen detection rate in summer and autumn of 2020 gradually increased to the pre-epidemic levels in 2021 after decreasing compared with 2019, whereas the pathogen detection rate in winter of 2020 was significantly higher (P < 0.05) compared with 2018 and 2019 and gradually decreased in 2021, the conclusion that further corroborates the seasonal prevalence of severe pneumonia.

In summary, the pathogenic characteristics of severe pneumonia in children in PICU are correlated with the age of children and the season of onset, and the prevalence varies in different regions and hospitals. The pathogens of pediatric patients with severe pneumonia in PICU commonly involves bacteria and viruses. As the age of children grows, the detection rate of bacteria shows a decreasing trend, and the pathogenic spectrum gradually changes from bacteria to mycoplasma and viruses, and the number of mixed infections gradually increase. Rational selection of antimicrobial drugs needs to consider pathogenic characteristics of different age, gender, and onset season in clinical practice. In addition, the prevalence of drug-resistant pathogens in healthcare settings plays a crucial role in determining the appropriate choice of antibiotics for empirical therapy. High rates of drug resistance can occur due to several factors, including excessive or inappropriate antibiotic use, inadequate infection control measures, and the transmission of resistant strains within healthcare facilities. When selecting an empirical antimicrobial regimen, factors such as the severity of the infection, the individual patient’s characteristics, local epidemiological data and the rate of drug-resistant pathogens should be considered. Therefore, it is important to grasp the changes of drug resistance according to the characteristics of the pathogenic distribution of the hospital and the clinical departments in this region, and formulate clinical anti-infection treatment plans according to drug sensitivity results, and rational use of antibacterial drugs to improve clinical efficacy and reduce the generation of bacterial drug resistance.

## Limitation

One limitation of this study is the reliance on culture-based methods to identify the bacterial etiology. It should be noted that culture has been reported to have a low sensitivity in detecting bacterial pathogens. Therefore, it is possible that the etiological results obtained from the initial culture may not accurately represent the true bacterial pathogens present in the patients. Alternative diagnostic methods with higher sensitivity, such as molecular techniques or advanced microbiological assays, could provide more accurate identification of bacterial etiology. Future studies should consider incorporating these methods to enhance the accuracy of bacterial pathogen detection and improve the understanding of the underlying causes of the infections. In our study, we acknowledge the presence of heterogeneous subjects, which could potentially affect the generalizability of the study results for practical application. To address the concern of generalizability, further research is warranted to validate and extend our findings to a broader population or different settings. This could involve larger sample sizes, diverse demographic characteristics, and multi-center studies to enhance the external validity of the results. Additionally, conducting comparative studies with other relevant populations could help establish the generalizability and applicability of the findings in practical settings.

## Conclusions

In this study, we analyzed the pathogenic and clinical characteristics of SP in PICU. Rational selection of antimicrobial drugs needs to consider pathogenic characteristics of different age, gender, and onset season in clinical practice. We aim to contribute to the existing knowledge on SP, provide valuable clinical insights, and guide clinicians in making informed decisions regarding empirical antimicrobial therapy for children with SP.

## Data Availability

The original contributions presented in the study are included in the article, further inquiries can be directed to the corresponding author.
